# Optimizing urinary tract infection outcomes: nitrofurantoin versus fluoroquinolones

**DOI:** 10.6026/973206300223777

**Published:** 2026-06-30

**Authors:** Shruti Vihang Brahmbhatt, Samarth Savaliya, Mahavirsingh Rajput, Archana Patel

**Affiliations:** 1 Department of Pharmacology, Shrimati Bhikhiben Kanjibhai Shah Medical Institute and Research Centre, Sumandeep Vidyapeeth Deemed to be University, Vadodara- 391760, Gujarat, India

**Keywords:** Urinary tract infection (UTI), nitrofurantoin, fluroquinolones, adverse drug reaction (ADR), antibiotic stewardship

## Abstract

The rising global prevalence of urinary tract infections and escalating antimicrobial resistance present a critical clinical challenge. Therefore, it is of interest to evaluate the clinical cure rates while minimizing adverse drug reactions (ADRs) and resistance, researchers studied 40 adult patients (80% female) divided into Group I (Nitrofurantoin) and Group II (Fluoroquinolones). Nitrofurantoin achieved a higher clinical cure rate (95%) with 4.75 times greater odds of success than fluoroquinolones (80%). The adverse reaction rate was significantly lower for nitrofurantoin (10% versus 26.7%), though both groups reported similar side effects. Thus, data shows the nitrofurantoin also maintained a lower failure rate (5% versus 15%), supporting its prioritization as a first-line empirical therapy.

## Background:

Urinary tract infections (UTIs) are among the most frequently occurring bacterial infections globally, primarily caused by uropathogenic Escherichia coli and other microorganisms like Klebsiella pneumoniae and Proteus mirabilis [1]. Lower urinary tract infections typically cause symptoms like painful and frequent urination [2]. Identifying the uropathogen through a urine culture with the presence of clinical symptoms remains the diagnostic gold standard [3]. Commonly used antibiotics include trimethoprim-sulfamethoxazole, nitrofurantoin and fluoroquinolones [4]. Nitrofurantoin is a synthetic antibiotic with potential side effects such as liver toxicity, nerve damage and lung injury primarily associated with long-term use [5]. Nitrofurantoin macrocrystals are utilized for the treatment and prevention of recurrent urinary tract infections [6]. Inside bacterial cells, nitrofurantoin is converted into reactive intermediates that inhibit ribosomal proteins and DNA [7]. Fluoroquinolones target bacterial DNA gyrase for both complicated and uncomplicated infections, but increased clinical use has led to rising microbial resistance [8]. Careful antibiotic selection is essential given the growing threat of antimicrobial resistance [9]. Therefore, it is of interest to compare the efficacy, safety and tolerability of nitrofurantoin and fluoroquinolones in patients with urinary tract infections at a tertiary care centre.

## Materials and Methodology:

## Study design and setting:

This study employed a prospective, observational (non-interventional) design and was conducted in Medicine, Surgery and Gynaecology wards of Dhiraj Hospital, a rural tertiary care teaching hospital affiliated with the S.B.K.S. Medical Institute & Research Centre under Sumandeep Vidyapeeth, Piparia, Vadodara. The study protocol was initiated only after receiving formal approval from the Institutional Ethics Committee (IEC) of Sumandeep Vidyapeeth.

## Participant enrolment:

The study population consisted of patients admitted to the Medicine, Surgery and Gynaecology wards who were prescribed either Nitrofurantoin or Fluoroquinolones for the treatment of a Urinary Tract Infection (UTI). Participants were enrolled using a simple randomization method. Prior to recruitment, each patient was provided with a detailed information sheet and a thorough verbal explanation of the study's purpose and methodology in their vernacular language. Written informed consent was obtained from all participants before any data were gathered.

## Data collection and ethical considerations:

Relevant clinical information was extracted from the patients' available case records and documented using a pre-designed, suitable data collection form. The parameters recorded included demographic details, prescription patterns and the total duration of hospital stay. To maintain strict participant confidentiality, personal identification was replaced with unique code numbers during the recording process.

## Assessment of outcomes:

Clinical progress was monitored throughout the hospitalization period and the specific condition of each patient was recorded at the time of discharge. The assessment of clinical cure and symptomatic improvement was documented using a standardized questionnaire. All collected data were systematically organized in a Case Record Form to ensure a comprehensive evaluation of the treatment outcomes within the rural clinical setting.

## Patient selection criteria:

## Inclusion criteria:

This study included adults (18+) of any gender hospitalized with urinary tract infections that required treatment with either nitrofurantoin or fluoroquinolones. All participants were enrolled only after providing voluntary, written informed consent.

## Exclusion criteria:

The study excluded individuals under the age of 18 as well as any patients who were unwilling to provide written informed consent.

## Sample size and sampling technique:

The study utilized a sample size of 40 participants, determined through a purposive sampling approach. Subjects were selected via simple randomization from the populations of the Medicine, Gynaecology and Surgical wards at Dhiraj General Hospital. Eligibility was strictly limited to patients diagnosed with and treated for Urinary Tract Infections (UTIs) during their admission.

## Results:

A total of 40 patients (N = 40) were evaluated in this study. The patient population was primarily young to middle-aged adults, with the highest concentration in the 31-45 years age bracket, which accounted for 47.5% (n = 19) of the participants. This was followed by the 18-30 years bracket at 40.0% (n = 16) and the 46-60 years bracket at 12.5% (n = 5). Regarding gender distribution, the study population showed a strong female predominance. Out of the 40 participants, 80.0% (n = 32) were female, while 20.0% (n = 8) were male (Figure 1 - see PDF). The prescription of the 40 participants was evenly split between two groups; 20 each, for Group I - Nitrofurantoin and Group II - Fluroquinolones. In group - II, Ciprofloxacin was the most commonly prescribed Fluroquinolone in 75% (n=15) participants. Followed by Norfloxacin in 20% (n=4) participants and Levofloxacin in 5%, i.e., a single patient. The remaining 20 participants were prescribed Nitrofurantoin. Out of 40 participants, 77.5% (n=31) received antibiotic via oral route, whereas the remaining 22.5% (n=9) received intravenous antibiotics. Dosing strategies varied according to the chosen agent. The most common dose prescribed was 100 mg, strictly associated with Nitrofurantoin, accounting for 50.0% (n = 20) of all prescriptions. For the Fluoroquinolones, a 400 mg dose was given to 25.0% (n = 10) of patients getting intravenous route of administration for drugs, while a 500 mg dose was prescribed to 22.5% (n = 9) of patients getting the drug orally. A higher dose of 750 mg was utilized in only a single case (2.5%). The frequency of drug administration was highly uniform across the study; almost all patients (97.5%, n = 39) were placed on a twice-daily (BD) regimen. Only 2.5% (n = 1) were prescribed a once-daily (OD) regimen (Figure 2 - see PDF). For the duration of the treatment, 20% (n=8) participants took the medications for 3 days, out of them 4 participants from Nitrofurantoin group and 4 from Fluroquinolone group. The rest 80% (n=32) took the medication for 5 days, 16 of them from Nitrofurantoin group and rest of the 16 from Fluroquinolone group (Table 1). The efficacy of prescribed medications was evaluated by comparing the symptoms at day of admission versus the day of follow up. Amongst both the groups, 92.5% (n=37) showed clinical improvement while clinical cure was seen in 87.5% (n=35) of the individuals. Amongst the Fluroquinolones group, 90% (n=18) showed clinical improvement and 80% (n=16) showed clinical cure while the rest 20% (n=4) had still persistent symptoms. In the Nitrofurantoin group, 95% (n=19) individuals showed clinical improvement and 95% (n=19) showed clinical cure while the rest 5% (n=1) had still persistent symptoms (Figure 3 - see PDF). Out of 40 individuals, 85% (n=34) did not report any kind of adverse drug reactions. While the rest 15% (n=6) showed adverse drug reactions with maximum cases of dizziness and headache, followed by nausea and vomiting. Ciprofloxacin showed 26.7% of ADR rate (4 out of 15 individuals); while Nitrofurantoin showed 10% ADR rate (2 out of 20 individuals). Meanwhile, Norfloxacin and Levofloxacin showed no ADR (Table 2).

## Discussion:

Urinary Tract Infections are one of the common infectious diseases with a prevalence rate varying between 3.14% and 19.87% due to regional differences in sanitation and healthcare access [9]. The increasing rate and resistance of antibiotics exerts a need to find an effective therapy for management of UTI. Our study found out that the predominant gender suffering from UTIs is female and the age group 18-35 with maximum number of cases which was consistent with the findings of a study done by Johny *et al.* [10]. The lower urinary tract anatomy and its location, being proximal to anus and reproductive organs in females contribute to increasing prevalence of UTI in them [11, 12]. To align with the Infectious Diseases Society of America (IDSA) 2025 guidelines and the findings of a study conducted by Luna *et al.* [13], our study aimed at observing the rates of clinical cure and clinical improvement in patients with UTI over the course of one week in Fluoroquinolones group and five days in Nitrofurantoin group. The results of the study suggested that clinical improvement was seen in 92.5% of the population and clinical cure was seen in 87.5% of the study population. This observation is corroborated by Alanazi *et al.* [14] who found that Nitrofurantoin had a clinical cure rate of 93.2% compared to Ciprofloxacin in a tertiary care setting. The failure of therapy rate for Fluroquinolones in our study was found to be 15%, corroborating to the Alanazi *et al.* study as well with a failure rate for Fluoroquinolones at 11.9% Chavda *et al.* [4] found a high sensitivity rate of 84.37% for E. coli isolates treated with Nitrofurantoin. Our clinical data supports this, with the Nitrofurantoin group achieving a 95% clinical cure rate. Furthermore, the stability of Nitrofurantoin is evidenced by Larkin *et al.* [15], in their meta-analysis the prevalence of resistance for Nitrofurantoin was seen only 6.9% which was similar to our findings with the resistance for Nitrofurantoin in 5% of the cases, evidently depicting a success rate for Nitrofurantoin as an antibiotic in the treatment of UTI. Beyond efficacy, the safety profile and adverse drug reactions (ADR) are the next major determinants of patient adherence and clinical success. In our study, participants with the Nitrofurantoin group had an ADR rate of 10% and the Fluroquinolones group had ADR rate of 26.7%, all ADRs were reported with usage of Ciprofloxacin. These results align with findings of Ahanjan *et al.* [16] where ADRs were observed in 44% of the patient receiving Ciprofloxacin while ADR rate of Nitrofurantoin group was 20%. Given Fluroquinolones are more effective in terms of tissue penetration, their systemic impact - including potential CNS side effects often leads to higher rates of discomfort. The above results statistically depicts that there are 4.75 times higher odds of achieving clinical cure for Nitrofurantoin compared to Fluroquinolones. Meanwhile, the odds of experiencing ADR with Nitrofurantoin group were 0.44 times less (reduced by more than half) in comparison to Fluroquinolones group. The findings of our study contribute to growing evidence of "Antibiotic Stewardship" - the systematic effort to educate and persuade clinicians to prescribe antibiotics more responsibly. The importance of stewardship is more pronounced in vulnerable populations. Datta *et al.* [17] reported that even in long-term care residents, where antibiotic exposure is high and Nitrofurantoin susceptibility remained preserved at 86.5%. This suggests that Nitrofurantoin is a resilient antibiotic that should be the cornerstone of empirical therapy protocols. By utilizing Nitrofurantoin for the majority of patients (as seen in our 95% cure rate), we can reduce the selective pressure that leads to the rise of multi-drug resistant organisms.

## Conclusion:

Nitrofurantoin showed clear clinical superiority for UTIs, achieving a 95% cure rate compared to 80% for fluoroquinolones. It also offered a safer profile, with an adverse reaction rate of just 10% versus 26.7% for fluoroquinolones. Thus, nitrofurantoin as the preferred empirical therapy to align with antibiotic stewardship and preserve fluoroquinolones.

## Advancement to knowledge:

This study advances clinical knowledge by demonstrating that in a rural tertiary care setting, Nitrofurantoin out-performs Fluoroquinolones for urinary tract infections (UTIs), achieving a 95% clinical cure rate compared to 80% for Fluoroquinolones. Additionally, it establishes a much safer tolerability profile, with Nitrofurantoin exhibiting a 10% adverse drug reaction (ADR) rate versus 26.7% for Ciprofloxacin. These findings provide critical, localized evidence supporting antibiotic stewardship protocols. They validate prioritizing Nitrofurantoin as a highly effective, resilient first-line empirical therapy, thereby preserving Fluoroquinolones for more severe, systemic infections and mitigating the escalation of antimicrobial resistance.

## Financial support and sponsorship:

Nil

## Figures and Tables

**Table 1:** Duration of prescribed drugs

**Duration of Treatment**	**Nitrofurantoin Group (n)**	**Fluoroquinolones Group (n)**	**Total (n)**	**Percentage (%)**
3 Days	4	4	8	20%
5 Days	16	16	32	80%
Total	20	20	40	100%

**Table 2:** The adverse drug reactions observed during the treatment

**Drug Name**	**Total Patients (n)**	**Patients with ADR (n)**	**ADR Rate (%)**
Nitrofurantoin	20	2	10.00%
Ciprofloxacin	15	4	26.70%
Norfloxacin	4	0	0.00%
Levofloxacin	1	0	0.00%
Total	40	6	15.00%
